# Urothelial toxicity of esketamine in the treatment of depression

**DOI:** 10.1007/s00213-020-05611-y

**Published:** 2020-07-26

**Authors:** Hannelore Findeis, Cathrin Sauer, Anthony Cleare, Michael Bauer, Philipp Ritter

**Affiliations:** 1 Klinik und Poliklinik für Psychiatrie und Psychotherapie, Universitätsklinikum Carl Gustav Carus an der Technischen Universität Dresden, Dresden, Germany; 2 King’s College London – Institute of Psychiatry, Denmark Hill, London, GB UK

**Keywords:** Esketamine, Affective disorder, Side effects, Urothelial toxicity, Depression

## Abstract

**Rationale:**

Ketamine is the first widely used substance with rapid-onset antidepressant action. However, there are uncertainties regarding its potential urothelial toxicity, particularly after repeated application. In the context of rising recreational ketamine use, severe side effects affecting the human urinary tract have been reported. It is assumed that ketamine interacts with bladder urothelial cells and induces apoptosis.

**Objectives:**

This study aimed to assess whether single or repeated doses of esketamine used in an antidepressant indication are associated with urinary toxicity.

**Methods:**

We included male and female inpatients with a current episode of depression and a diagnosis of recurrent depressive disorder, bipolar disorder or schizoaffective disorder according to ICD-10 criteria (*n* = 25). The esketamine treatment schedule involved a maximum of 3× weekly dosing at 0.25–0.5 mg/kg i.v. or s.c. The primary outcome was the change in urine toxicity markers (leukocytes, erythrocytes, protein and free haemoglobin). Description of demographic, clinical and laboratory data was conducted using means, standard deviations, frequencies and percentages. Changes in urinary toxicity markers over time were evaluated using linear mixed models with gender as a covariate.

**Results:**

The participants received an average of 11.4 (SD 8) esketamine treatments, and an average number of 11.2 (SD 8) urine samples were analysed over the course of treatment. Neither urinary leukocyte concentration (F(20; 3.0) = 3.1; *p* = 0.2) nor erythrocyte concentration (F(20;2.2) = 4.1; *p* = 0.2) showed a significant trend towards increase during the course of esketamine treatment. Similarly, free haemoglobin and protein concentrations, which were analysed descriptively, did not display a rise during treatment. There was a significant improvement in depression ratings after esketamine treatment (*p* < 0.001).

**Conclusions:**

This study is, to the best of our knowledge, the first to focus on urothelial toxicity of esketamine used in antidepressant indication and dose. The results indicate that the use of single or repeated doses of esketamine is unlikely to cause urothelial toxicity. The results are in need of confirmation as sample size was small.

**Electronic supplementary material:**

The online version of this article (10.1007/s00213-020-05611-y) contains supplementary material, which is available to authorized users.

## Introduction

Ketamine has been successfully used in anaesthesia and emergency medicine as a rapid-onset and short-duration analgesic and narcotic substance since the 1960s. It is primarily an *N*-methyl-D-aspartate (NMDA) receptor antagonist with additional effects on dopamine D2 (Kapur and Seeman 2001) and several opioid receptors (Kohrs and Durieux 1998), as well as an inhibitory effect on serotonin and norepinephrine reuptake (Krystal et al. 1999, 2006; Javitt 2007).

Multiple studies and clinical experience have shown that remission can be achieved in less than 50% of patients with commonly used antidepressant treatments (Rush et al. 2006; Undurraga and Baldessarini 2012) and that effective treatment is hampered by a delayed onset of action (Thompson 2002; Insel and Wang 2009; Harmer et al. 2017). Ketamine and its S-isomer esketamine, which is assumed to have a greater analgesic and anaesthetic activity with less psychotomimetic effects than the racemic mixture or its R-isomer (Muller et al. 2016), are the first widely used substances with rapid-onset antidepressant action. Their efficacy in subanaesthetic doses has been established in numerous studies (Romeo et al. 2015; Muller et al. 2016) including both single-dose (Berman et al., 2000; Zarate et al. 2006) and repeated-dose application (Rasmussen et al. 2013; Murrough et al. 2013b; Shiroma et al. 2014). However, doubts regarding its potential urothelial toxicity, particularly after repeated application, remain (Short et al. 2018).

In the context of rising recreational ketamine use, severe side effects affecting the human urinary tract have been reported, such as frequent and painful urination, haematuria, suprapubic pain and ulcerative cystitis (Shahani et al. 2007; Ho et al. 2010; Kalsi et al. 2011; Middela and Pearce 2011; Morgan and Curran 2012). Both emergency cystectomy and an association with urothelial carcinoma (Oxley et al. 2009; Morgan and Curran 2012) have been reported in both recreational (Chu et al. 2008; Middela and Pearce 2011; Reinhardt and Fode 2014) and therapeutic use (Shahzad et al. 2012) of ketamine. In addition, studies on the side effects of ketamine treatment for chronic pain confirmed that urinary symptoms can emerge after repeated administration in high doses (Storr & Quibell, 2009; Persson 2010).

Although the precise mechanism by which an excessive amount of ketamine or its metabolites are associated with cystitis is not known for certain, it is assumed that ketamine interacts with bladder urothelial cells and interstitial tissues and induces apoptosis (Chu et al. 2008; Wood et al. 2011; Tsai and Kuo 2015; Baker et al. 2016), an effect that appears to be mediated by the NMDA receptor (Takadera et al. 2006; Wang et al. 2006), triggering prolonged elevation of cytosolic calcium concentrations. Ex vivo studies have shown that a cytosolic concentration of ketamine of 1 mmol/l in non-immortalized human urothelial cells generates dose-related cytotoxic effects by inducing the intrinsic apoptotic pathway (Baker et al. 2016). Moreover it appears that ketamine or its metabolites induce microvascular changes in the bladder and possibly the kidney causing an autoimmune reaction against the bladder urothelium and submucosa (Chu et al. 2008).

The most stable urinary marker of early ketamine-induced cystitis has been an increase in urinary erythrocytes and haemoglobin (Shahani et al. 2007; Meng et al. 2013; Jhang et al. 2015; Yang et al. 2015).

Despite the potential urological toxicity and the numerous studies regarding the general side effects of clinical ketamine use, there have, to the best of our knowledge, been no published investigations on the urothelial toxicity of ketamine and esketamine when used in antidepressant dose and indication (Hashimoto 2016; Xu et al. 2016; Daly et al. 2017; Gálvez et al. 2018; Citrome et al. 2020; Kryst et al. 2020). In a recent systematic review on ketamine-associated side effects, Short et al. detailed that only 5 out of 60 therapeutic studies assessed any urinary tract symptoms (subjective urinary complaints and drug/pregnancy screenings) (Short et al. 2018). None of the published studies investigated urinary clinical and laboratory parameters as a marker for lower urinary tract damage. It was shown that the most common acute side effects to be reported were headache, dizziness, dissociation, elevated blood pressure and blurred vision, most of which were reported to have resolved shortly after dose administration (Berman et al. 2000; Zarate et al. 2006; Diazgranados et al. 2010; Trial et al. 2012; Carlson et al. 2013). Moreover, relatively few studies have examined the long-term safety of repeated ketamine treatment (George et al., 2017; Murrough et al., 2013a, b).

This study aimed to assess whether single or repeated doses of esketamine used for an antidepressant indication are associated with urinary toxicity as determined by direct laboratory assessment of urinary toxicity markers collected prior to, during and after treatment.

## Methods

### Study design and participants

The study was approved by the institutional review board of the Medical Faculty of the Technische Universität Dresden (IRB00001473 and IORG0001076) granting the retrospective, anonymized analysis of data obtained in routine clinical practice without individual patient consent.

We included male and female inpatients with a current moderate or severe episode of depression and a diagnosis of single or recurrent depressive disorder (ICD-10:F32, F33), bipolar disorder (ICD-10: F31) or schizoaffective disorder (ICD-10: F25) according to ICD-10 criteria. Only patients who had received at least one treatment with esketamine (administered subcutaneously or intravenously) in an antidepressant indication and dose (0.25–0.5 mg/kg body weight) during the period of March 2017–June 2019 were included in the analysis. The esketamine treatment schedule involved a maximum of 3× weekly dosing (Ritter et al. 2020). We selected only those with data on at least one urine sample prior to and one after completing esketamine treatment. The primary outcome was the change in urine toxicity markers (leukocytes, erythrocytes, protein and free haemoglobin).

### Assessments/materials

Standard midstream urine samples were collected in sterile polypropylene beakers prior to initiation, during and following esketamine treatment. The last follow-up was 1 day after receiving the last esketamine treatment. In addition, severity of depression was assessed before, during and at the end of esketamine treatment using the Beck Depression Inventory (BDI II) (Beck et al. 1961), a validated self-rating instrument.

The following urinary parameters were evaluated: erythrocyte concentration in Mpt/l (normal value < 23 Mpt/l), leucocyte concentration in Mpt/l (normal value < 25 Mpt/l), free haemoglobin in nmol/l (normal value 0 mmol/l) and protein in g/l (normal value < 0.15 g/l). Whereas leucocytes, erythrocytes and protein have a certain cut-off value under which the parameters are non-pathological in human urine, haemoglobin always indicates a pathological process of the urinary tract (for common causes of haematuria, haemoglobinuria, leucocyturia and proteinuria, see supplement 2).

All analyses were conducted at the biochemical laboratory of the University Hospital TU Dresden. Urinary samples were processed using the manufacturer’s protocol. Erythrocytes (detection limit: 10 Mpt/l) and leucocytes (detection limit: 25 Mpt/l) were assessed using UF-5000 (Sysmex Europe GmbH) for urine flow cytometry according to the manufacturers’ protocol. Free haemoglobin (detection limit: 18.6 nmol/l) and protein (detection limit: 0.15 g/l) were assessed using UC-3500 (Sysmex Europe GmbH) with wet chemistry test strips according to the manufacturers’ protocol. The analysis of the urine parameters was completed within 2 h of sample collection.

In addition, we recorded the patient’s age at the time of the first treatment, their gender, number of total esketamine treatments, diagnosis, comorbidities and the dates of the urine samples.

### Analysis

Description of demographic, clinical and laboratory data was conducted using means, standard deviations, frequencies and percentages. The laboratory values that we examined are prone to be affected by other patient factors, for example, urinary tract infection or menstruation. Therefore, in order to control for potential bias, we corrected the data by removing outliers defined as values outside the individual 3* interquartile range (IQR). The individual IQR was defined on the basis of all data points for a given participant.

In addition, we conducted analyses using three further approaches: removing outliers defined as values outside of the collective 1.5* IQR, removing outliers defined as values outside the collective 3* IQR and not removing outliers at all (see supplement 1). The collective IQR was determined by all data points for the entire sample.

Changes in urinary toxicity markers over time were evaluated using linear mixed models with gender as a covariate. Outcome measures were urinary erythrocyte and leukocyte concentration.

Free urinary haemoglobin and protein concentrations were analysed descriptively, in order to see whether the number of individuals with detectable levels of either parameter changed during esketamine treatment. Significance level was set at 5%. All analyses were conducted using IBM SPSS Statistics for Windows, Version 25.

The primary hypothesis was that no significant gradient in any of the four toxicity markers would be observed.

### Outcome

#### Participants

Participants’ mean age was 49 years (SD 15). A total of 10 male and 15 female patients receiving esketamine had complete data sets for analysis. One participant was excluded due to bacterial cystitis at study entry. Sixteen of the patients suffered from unipolar depression, 7 from bipolar depression and 2 had a schizoaffective disorder without psychotic symptoms at time of treatment. The participants received an average of 11.4 (SD 8) esketamine treatments (0.5 mg/kg). Two patients received only one infusion; the reason for discontinuation was poor subjective tolerability in both cases. Three patients received 20 or more (maximum 34) infusions. An average number of 11.2 (SD 8) urine samples were analysed over the course of treatment (Table 1).

**Table 1:** Clinical and demographic data for all (*n* = 25) participants

Clinical & Demographic data		
		Mean (SD)
Age		49 (15)
Number of Esketamine-treatments		11.4 (8)
BDI II (all patients) pre treatment		30.9 (13.25)
	unipolar	33
	bipolar	25
	schizoaffective	36
BDI II (all patients) post treatment		20.9 (13.75)
	unipolar	24
	bipolar	12
	schizoaffective	25
Number of urine samples per patient		11.2 (8)
		Percentages
Gender	male	40%
	female	60%
Primary Diagnosis	unipolar	64%
	bipolar	28%
	schizoaffective	8%
	Atrial Fibrillation	4%
	Peripheral Polyneuropathy	4%
	Obstructive Sleep Apnoea	4%
	Pulmonary Sarcoidosis	4%
Psychiatric Comorbidities (including Substance Abuse)	Emotionally Instable Personality Disorder	16%
	Current Alcohol Misuse	16%
	Post Traumatic Stress Disorder	8%
	Somatoform Disorder	4%

#### Urothelial toxicity

**Fig. 1:**
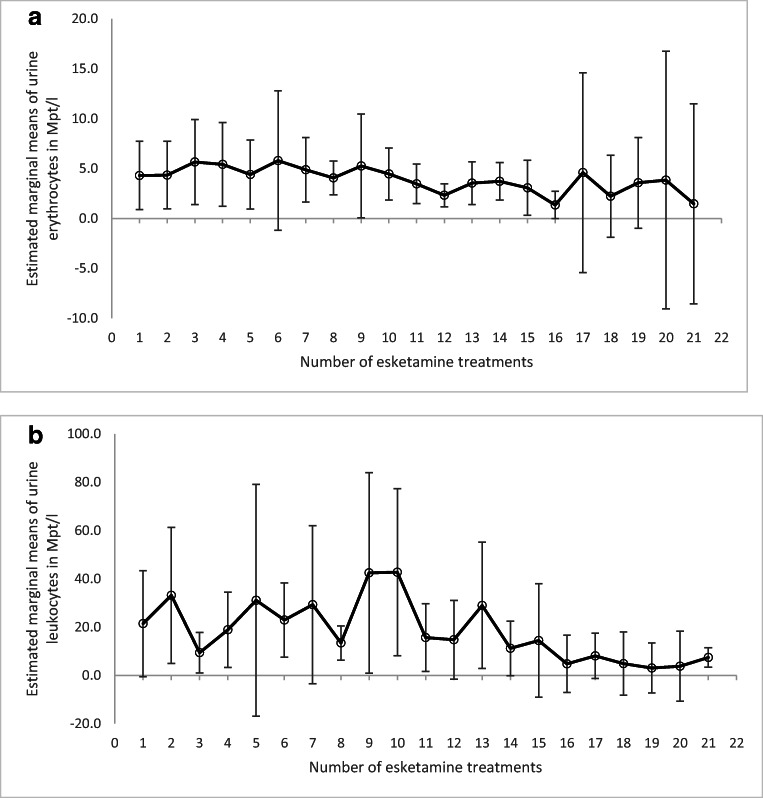
**a** Estimated marginal means of urine erythrocytes in Mpt/l prior to esketamine administration over the time course of multiple (*x*-axis) esketamine treatments, removing individual outliers for each patient of the 3* IQR. Values corrected for gender. Error bars: standard deviation. **b** Estimated marginal means of urine leukocytes in Mpt/l prior to esketamine administration over the time course of multiple (*x*-axis) esketamine treatments, removing individual outliers for each patient of the 3* IQR. Values corrected for gender. Error bars: standard deviation

No significant gradient in any of the four toxicity markers was observed. Neither urinary leukocyte concentration (F(20; 3.0) = 3.1; *p* = 0.2) nor erythrocyte concentration (F(20;2.2) = 4.1; *p* = 0.2) showed a significant trend to increase during the course of esketamine treatment (Fig. 1a, b). Mean leukocyte concentration and erythrocyte concentration were both significantly elevated in women compared with men (leukocytes: F(1;101.4) = 41.3; *p* < 0.001; erythrocytes: F(1;16.4) = 5.4; *p* = 0.03), but the interaction between timepoint and gender showed no significant difference over the course of time (leukocytes: F(20;2.99) = 2.8; *p* = 0.2; erythrocytes: F(20;2.2) = 1.7; *p* = 0.4). Outlier removal had to be conducted for 5 participants: the first with 1 leucocyte value and 3 erythrocyte values, the second with 2 leucocyte values, the third with 1 erythrocyte value, the fourth with 1 leucocyte value and 1 erythrocyte value and the fifth with 1 erythrocyte value. In total, 10 outlier values were removed.

No changes in the gradient of toxicity markers were observed for all four approaches to removing outliers (see supplement 1 Fig. 1a - 7b for the other approaches to outlier correction).

**Fig. 2:**
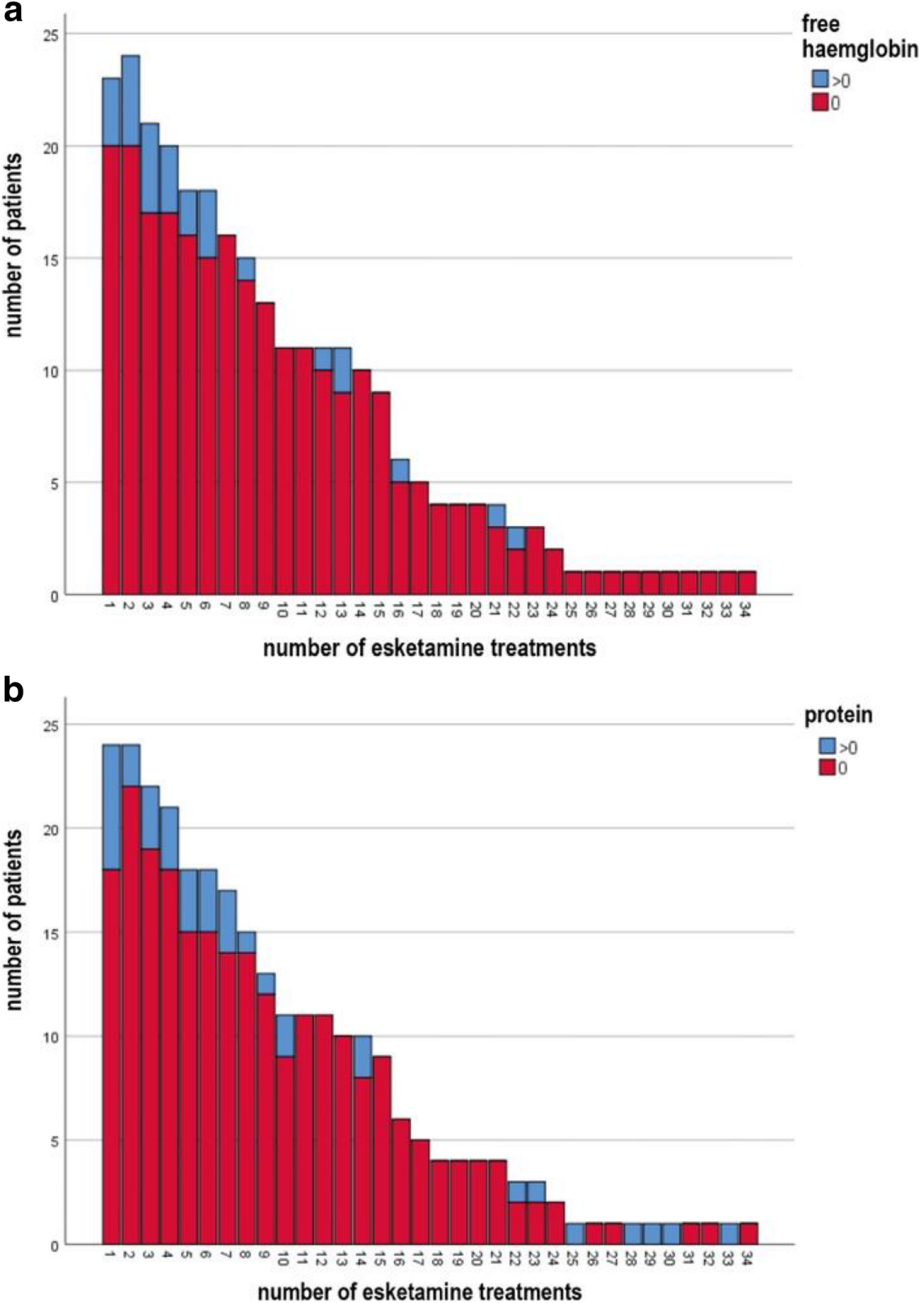
**a** Urinary free haemoglobin concentration (blue = positive, > 0 nmol/l; red = negative, 0 nmol/l) prior to esketamine administration over the time course of multiple esketamine treatments. **b** Urinary protein concentration (blue = positive, > 0 g/l; red = negative, 0 g/l) prior to esketamine administration over the time course of multiple esketamine treatments

Free haemoglobin and protein were analysed descriptively (Fig. 2a, b); there was no observable increase in the number of individuals with detectable levels of either parameter.

### Mood outcomes

There was a significant change in depression ratings after esketamine treatment (*p* < 0.001). The mean BDI II value before starting the esketamine treatment was 31 (SD 13). After completing the esketamine infusions, BDI II decreased to a mean value of 20 (SD 14). The largest reduction of BDI II values occurred in the patients suffering from bipolar depression (BDI II pretreatment 25 vs. BDI II posttreatment 12).

## Discussion

The absence of change from baseline in the four measured urinary parameters strongly suggests that neither single nor repeated infusions of esketamine in an antidepressant dose (0.25–0.5 mg/kg) cause short-term urothelial damage. Our sample included patients who had received up to 34 infusions without an indication of rising toxicity markers from which one could tentatively infer that there is also no cumulative urothelial injury when doses are administered on alternate days and no more frequent than 3× week.

Several limitations need to be considered.

Potential urothelial toxicity was investigated by examining urinary leukocytes, erythrocytes, protein and free haemoglobin as indirect markers of urothelial toxicity. Urinary erythrocytes and haemoglobin in particular are both well-established markers of acute ketamine-induced urothelial toxicity (Meng et al. 2013; Jhang et al. 2015; Yang et al. 2015). More subtle preliminary stages of urothelial damage may however not be accompanied by a rise in these markers and therefore not revealed in this study.

Moreover, our results are restricted to the immediate toxicity effects. Although not suggested by prior animal and human research, no conclusions regarding delayed toxicity can be drawn. Therefore, further research regarding long-term and delayed effects of esketamine as an antidepressant need to be investigated.

Although the sample size (*n* = 25) is moderate, there is no numerical trend towards an increase in toxicity markers within the 280 analysed urine samples that would suggest that the negative result may be due exclusively to a lack of power. Nevertheless, the results are in need of confirmation ideally with a larger sample.

This sample may not be representative of the patient population as a whole because patients with known uncontrolled hypertension, impaired renal function (eGFR < 30), severe substance abuse or previous urothelial pathology are excluded from esketamine treatment for safety reasons. The results should therefore not uncritically be extrapolated to patients with urological comorbidities or known substance abuse. Since no patients were receiving other treatments with potential urothelial toxicity (such as radiation or chemotherapy), no inferences regarding potential additive toxic effects can be made.

Although these data cannot inform considerations regarding any dose-response effects in the causation of urothelial damage, it would seem reasonable to conclude that, in the context of current preclinical and clinical studies, toxicity is likely dose dependent and does not occur below a certain threshold. If esketamine and ketamine become established treatments in psychiatry possibly also using higher doses or more frequent dosing regimens than currently in use it will be essential to establish a safe dosing range in future studies.

## Conclusion

This study is, to the best of our knowledge, the first to focus on laboratory and biological markers of urothelial toxicity of esketamine used in antidepressant indication and dose. The sample included inpatients, with moderate or severe depression, and used a naturalistic study design. The results indicate that the use of single or repeated doses of esketamine at 0.25–0.5 mg/kg is unlikely to cause urothelial toxicity.

## Electronic supplementary material

ESM 1 (RTF 2605 kb)
