# Supplementary material for: Urothelial toxicity of esketamine in the treatment of depression
Source: Psychopharmacology (Berl). 2020 Jul 26;237(11):3295–302. doi: 10.1007/s00213-020-05611-y (PMC7561544; doi:10.1007/s00213-020-05611-y)
Supplement: Supplementary file 1 — (RTF 2605 kb) [file 213_2020_5611_MOESM1_ESM.rtf]

Supplement

Urothelial Toxicity of Esketamine in the Treatment of Depression


Journal:
PSYCHOPHARMACOLOGY

Authors: 
Hannelore Findeis1
Cathrin Sauer1 
Anthony Cleare2
Michael Bauer1
 Philipp Ritter1

Affiliation: 
1.	Klinik und Poliklinik für Psychiatrie und Psychotherapie, Universitätsklinikum Carl Gustav Carus an der Technischen Universität Dresden, Dresden, Deutschland
2.	King's College London – Institute of Psychiatry, Denmark Hill, London, GB


Corresponding Author: 
Philipp Ritter 
Department of Psychiatry, Universitätsklinikum Carl Gustav Carus an der Technischen Universität Dresden, Dresden Germany; philipp.ritter@ukdd.de; tel.: +49 (0)351 458-18763; fax.: +49 (0)351 458-5356


Supplement 1

No significant gradient in any of the four toxicity markers was observed. Neither urinary leukocyte concentration (F(20; 6,42)=1.9; p=0.2) or erythrocyte concentration (F(20,2; 18)=2.9; p=0.3)  showed a significant tendency to increase during the course of esketamine-treatment. Similarly free haemoglobin and protein concentrations, which were analysed descriptively, did not display an increase during treatment. 
Additionally to removing individual outliers for each patient of the 3* Interquartile range (IQR) there was no significant gradient when removing outliers of the 1.5* IQR, the 3* IQR or when not removing outliers at all. Also there were no differences depending on diagnosos (Figure 1a-3b) or gender differences (Figure 4a-6b). by applying any of these approaches of correcting data regarding outliers in order to control for potential bias.
When removing outliers defined as values outside of the 1.5* IQR, there was no significant change in urinary leucocytes nor in erythrocyte concentration during esketamine treatment (Figure 1a+b and 4a+b). The same held true for the 3* IQR (Figure 2a+b and 5a+b) and not removing outliers at all (Figure 3a+b and 6a+b).
No significant gradient in any of the four toxicity markers depending on gender differences (figure 7a+b) was observed. 


Figure 1a, estimated marginal means of urine erythrocytes in Mpt/l over the time course of multiple (x-axis) esketamine treatments; removing outliers defined as values outside of the 1.5* IQR. Values corrected for diagnosis. Error bars: standard deviation.


Figure 1b, estimated marginal means of urine leukocytes in Mpt/l over the time course of multiple (x-axis) esketamine treatments; removing outliers defined as values outside of the 1.5* IQR. Values corrected for diagnosis. Error bars: standard deviation.


Figure 2a, estimated marginal means of urine erythrocytes in Mpt/l over the time course of multiple (x-axis) esketamine treatments; removing outliers defined as values outside of the 3* IQR. Values corrected for diagnosis. Error bars: standard deviation.


Figure 2b, estimated marginal means of urine leukocytes in Mpt/l over the time course of multiple (x-axis) esketamine treatments; removing outliers defined as values outside of the 3* IQR. Values corrected for diagnosis. Error bars: standard deviation.


Figure 3a, estimated marginal means of urine erythrocytes in Mpt/l over the time course of multiple (x-axis) esketamine treatments; not removing outliers at all. Values corrected for diagnosis. Error bars: standard deviation.


Figure 3b, estimated marginal means of urine leukocytes in Mpt/l over the time course of multiple (x-axis) esketamine treatments; not removing outliers at all. Values corrected for diagnosis. Error bars: standard deviation.


Figure 4a, estimated marginal means of urine erythrocytes in Mpt/l over the time course of multiple (x-axis) esketamine treatments; removing outliers defined as values outside of the 1.5* IQR. Values corrected for gender. Error bars: standard deviation.


Figure 4b, estimated marginal means of urine leukocytes in Mpt/l over the time course of multiple (x-axis) esketamine treatments removing outliers defined as values outside of the 1.5* IQR. Values corrected for gender. Error bars: standard deviation.


Figure 5a estimated marginal means of urine erythrocytes in Mpt/l over the time course of multiple (x-axis) esketamine treatments; removing outliers defined as values outside of the 3* IQR. Values corrected for gender. Error bars: standard deviation.


Figure 5b, estimated marginal means of urine leukocytes in Mpt/l over the time course of multiple (x-axis) esketamine treatments removing outliers defined as values outside of the 3* IQR. Values corrected for gender. Error bars: standard deviation.


Figure 6a, estimated marginal means of urine erythrocytes in Mpt/l over the time course of multiple (x-axis) esketamine treatments; not removing outliers at all. Values corrected for gender. Error bars: standard deviation.


Figure 6b, estimated marginal means of urine leukocytes in Mpt/l over the time course of multiple (x-axis) esketamine treatments; not removing outliers at all. Values corrected for gender. Error bars: standard deviation.


Figure 7a, estimated marginal means of urine erythrocytes in Mpt/l) over the time course of multiple (x-axis) esketamine treatments; removing individual outliers for each patient of the 3* IQR. Values corrected for gender. Error bars: standard deviation.


Figure 7b, estimated marginal means of urine leukocytes in Mpt/l over the time course of multiple (x-axis) esketamine treatments; removing individual outliers for each patient of the 3* IQR. Values corrected for gender. Error bars: standard deviation.


Supplement 2
Haemoglobinuria can be caused by an intravasal haemolysis as a reaction to incompatible blood tranfsuion, haemolytic crisis, toxicity, tumour, inflammations or mechanical damage. Leucocyturia is mostly caused by cystitis  and pyelonephritis, but also by inflammations and mechanical damage. Erythrocyturia as a form of haematuria can be distinguished in pre-, renal or post-renal erythrocyturia. It may be caused by anticoagulation, haemorrhagical diathesis, glomerulonephritis, pyelonephritis, renal infarction, renal tuberculosis, traumata, tumour, cystitis or urolithiasis.
